# A multi-branch network to detect post-operative complications following hip arthroplasty on X-ray images

**DOI:** 10.3389/fbioe.2023.1239637

**Published:** 2023-09-28

**Authors:** Sijia Guo, Jiping Zhang, Huiwu Li, Jingwei Zhang, Cheng-Kung Cheng

**Affiliations:** ^1^ School of Biomedical Engineering, Shanghai Jiao Tong University, Shanghai, China; ^2^ Engineering Research Center for Digital Medicine of the Ministry of Education, Shanghai Jiao Tong University, Shanghai, China; ^3^ Department of Orthopaedics, Ninth People’s Hospital, Shanghai Jiao Tong University School of Medicine, Shanghai, China

**Keywords:** post-operative complications, total hip arthroplasty, deep learning, X-ray, multi-branch network, domain knowledge

## Abstract

**Background:** Postoperative complications following total hip arthroplasty (THA) often require revision surgery. X-rays are usually used to detect such complications, but manually identifying the location of the problem and making an accurate assessment can be subjective and time-consuming. Therefore, in this study, we propose a multi-branch network to automatically detect postoperative complications on X-ray images.

**Methods:** We developed a multi-branch network using ResNet as the backbone and two additional branches with a global feature stream and a channel feature stream for extracting features of interest. Additionally, inspired by our domain knowledge, we designed a multi-coefficient class-specific residual attention block to learn the correlations between different complications to improve the performance of the system.

**Results:** Our proposed method achieved state-of-the-art (SOTA) performance in detecting multiple complications, with mean average precision (mAP) and F1 scores of 0.346 and 0.429, respectively. The network also showed excellent performance at identifying aseptic loosening, with recall and precision rates of 0.929 and 0.897, respectively. Ablation experiments were conducted on detecting multiple complications and single complications, as well as internal and external datasets, demonstrating the effectiveness of our proposed modules.

**Conclusion:** Our deep learning method provides an accurate end-to-end solution for detecting postoperative complications following THA.

## 1 Introduction

Total hip arthroplasty (THA) is a common surgical procedure used to treat end-stage hip diseases (Learmonth et al., 2007; Ferguson et al., 2018), such as osteoarthritis. While THA is generally safe and effective, postoperative complications, including periprosthetic joint infections, dislocation, aseptic loosening, and periprosthetic fractures, often arise, leading to failure of the prosthesis (Healy et al., 2016; Kelmer et al., 2021; Patel et al., 2023). The identification of these complications typically relies on plain radiographs (Awan et al., 2013; Thejeel and Endo, 2022), which can be subjective, time-consuming, and prone to misdiagnosis. Therefore, a more accurate and timely method for detecting postoperative complications would help to optimize treatment planning and improve patient outcomes.

Recent advances in artificial intelligence have shown great promise in medical imaging analysis. Deep learning has been successfully applied to various medical imaging tasks, such as breast cancer screening (Shen et al., 2021), detecting skin lesions (Soenksen et al., 2021; Wu et al., 2022), osteoarthritis recognition (Thomas et al., 2020; Bayramoglu et al., 2021), and brain tumor segmentation (Grøvik et al., 2020). While several studies have made progress in recognizing individual complications, such as aseptic loosening (Shah et al., 2020; Lau et al., 2022; Loppini et al., 2022; Rahman et al., 2022) or dislocation (Rouzrokh et al., 2021), using deep learning methods, significant challenges remain in automatically identifying THA complications on X-ray images. These challenges include a lack of publicly available data, noisy and scattered features on medical image data, and the need to identify multiple complications simultaneously.

To address these challenges, this study introduces a novel deep learning method to automatically identify complications following THA on X-ray images. This study constructed a large dataset of hip revision cases with multi-label labelling of complications based on medical history data and radiographic findings. The performance of various state-of-the-art (SOTA) image classification models was evaluated using the dataset, with ResNet18 being used as a baseline. A novel multi-branch network model was then proposed that achieved better performance than alternative models at identifying both multi-label and single-task complications.

In summary, our work aims to provide a more effective and accurate method for identifying multiple complications following THA, leading to improved patient outcomes. Our main contributions include:

1. A dataset containing 443 X-ray images of hip prosthesis failures with multiclass annotation of postoperative complications following THA.
2. A multi-scale and multi-level network model for identifying post-THA complications on X-ray images.
3. An investigation of the effectiveness of domain prior knowledge fusion for identifying multi-category complications after THA surgery.

## 2 Methods

### 2.1 Overview of proposed architecture

**FIGURE 1:**
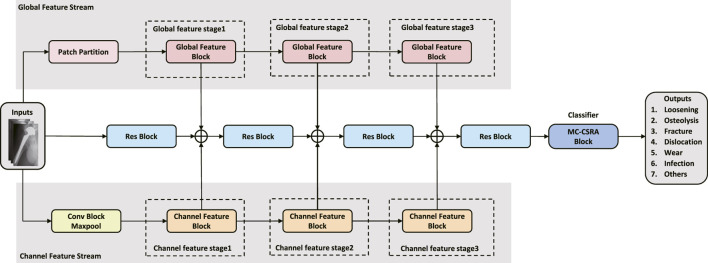
Architecture of the proposed model. The model features a ResNet18 backbone for initial feature extraction, and introduces Channel and Global Feature Flows for multi-scale feature extraction. Additionally, the MC-CSRA module is incorporated for learning label correlations, which can improve the accuracy of detecting complications.

As depicted in Figure 1, this study presents a multi-branch neural network for the assessment of complications after hip arthroplasty on X-ray images. Our network uses ResNet18 as the backbone, facilitating the initial extraction of features from input images through a deep convolutional neural network with residual connections (He et al., 2016). Two additional branches were also integrated, a channel feature stream and a global feature stream, to extract multi-scale features. To boost the accuracy, we also designed a multi-coefficient class-specific residual attention (MC-CSRA) block, which aimed to fuse domain knowledge by learning correlations between labels.

### 2.2 Global feature stream

**FIGURE 2:**
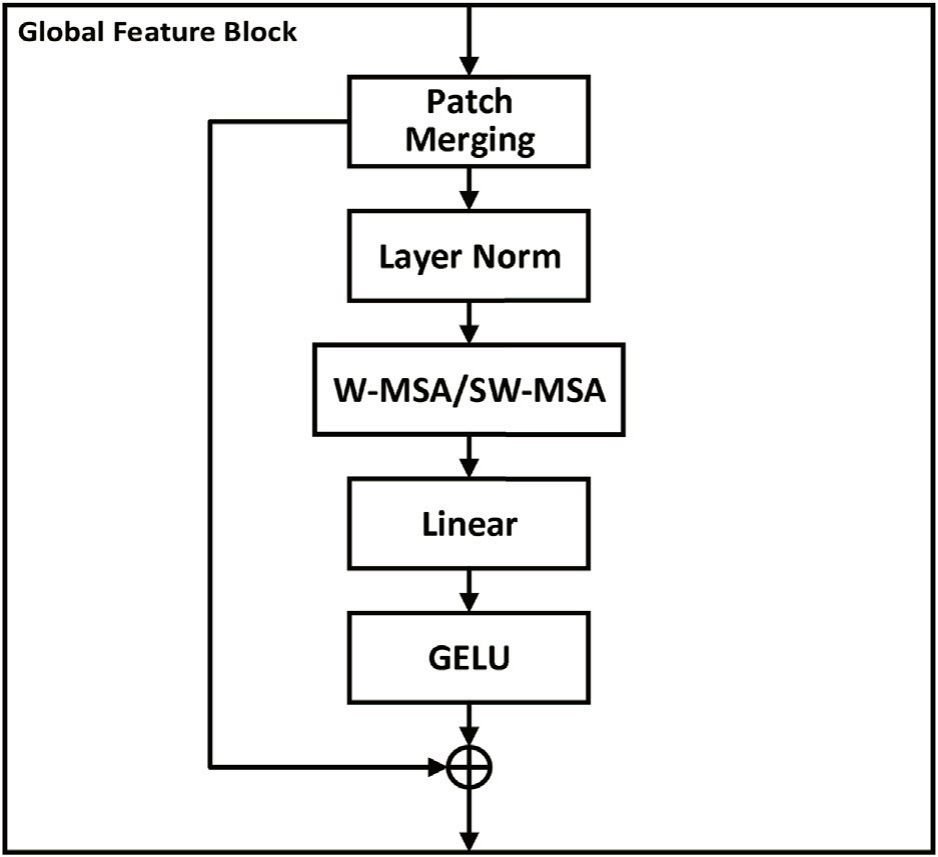
Global feature block. Each block processes fixed-size, non-overlapping patches of the input image, running through Patch Merging, Layer Norm, Window-based Multi-head Self-Attention (W-MSA) or Shifted Window-based Multi-head Self-Attention (SW-MSA), Linear, and GELU modules. This structure enhances the extraction of broad feature patterns and is designed to capture spatial relationships between patches, enabling a holistic understanding of the image and improving the model’s diagnostic capabilities.

As shown in Figure 2, a global feature stream was added to the network to allow global features to be extracted. The input image is first divided into fixed-size, non-overlapping patches using a 2D convolutional layer, filled as needed, and finally flattened and normalized. The output of the patch partition is then passed through three successive global feature blocks to extract the feature. The Swin transformer (Liu et al., 2021) uses the shift window to allow the model to capture the spatial relationships between adjacent patches, which can further improve the ability of the model to extract global features. Inspired by the Swin transformer, we designed global feature blocks that share a similar structure, encompassing Patch Merging, Layer Norm, Window-based Multi-head Self-Attention (W-MSA) or Shifted Window-based Multi-head Self-Attention (SW-MSA), Linear, and GELU modules. The global feature block can be formulated as:

$$f_{GF}\left(x\right)=LN\left(x+M\left(x\right)\right)MSA\left(x\right)+x,$$ (1)

where *x* is the input tensor, LN is the Layer Norm module, M is the Patch Merging module, and MSA is the W-MSA/SW-MSA module. Similar to the Swin transformer, the formula of the W-MSA/SW-MSA module is expressed as follows:

$$MSA\left(Q,K,V\right)=SoftMax\left(\frac{QK^{T}}{\sqrt{d_{k}}}\right)V,$$ (2)

where *Q*, *K*, and *V* are the query, key, and value matrices, respectively, and *d*_*k*_ is the dimension of the key vectors.

### 2.3 Channel feature stream

The channel feature stream is an essential component of the proposed method for extracting channel-level features from the input image. Initially, the input image is passed through the Conv Block and Maxpool layers, generating an initial feature map. Subsequently, the output is fed into a three-stage channel feature block, where a new feature map is extracted at each stage, emphasizing the important channels.

**FIGURE 3:**
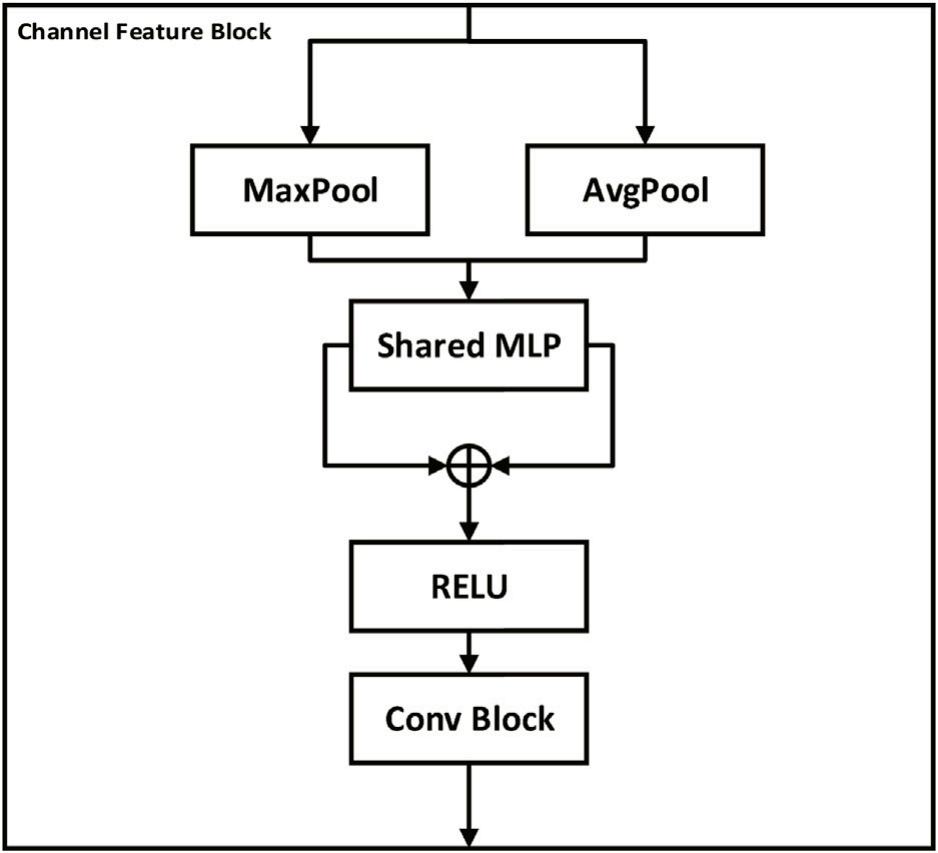
Channel feature block. The block consists of Conv, Maxpool layers, and a multi-layer perceptron (MLP) to extract channel-level features from the input image. Pooling layers (MaxPool and AvgPool) feed into the shared MLP, producing two attention maps which are added together and then passed through a sigmoid function to generate a final attention map. This attention map is element-wise multiplied with the input feature map to highlight important channels and thus enhance channel-wise relationships, thereby promoting more effective feature extraction and interpretation by the model.

Similar to the channel attention module (Woo et al., 2018), the channel feature block is designed to enhance the channel-wise relationships of feature maps by adaptively recalibrating feature responses. As shown in Figure 3, the channel feature block consists of two pooling layers (MaxPool and AvgPool) and a shared multi-layer perceptron (MLP) with two convolutional layers. The output of the pooling layers is fed into the shared MLP, which learns a channel-wise attention map by weighting the feature responses. The two attention maps obtained from the two pooling layers are added and passed through a sigmoid activation function to obtain the final attention map. The attention map is then multiplied element-wise with the input feature map to generate the output feature map. The formula for the channel feature block can be expressed as follows:

$$f_{CF}\left(X\right)=\sigma \left(g\left(\frac{1}{H\times W}\overset{H}{\underset{i=1}{\sum }}\overset{W}{\underset{j=1}{\sum }}x_{i,j}\right)W_{2}\left(\delta \left(W_{1}X\right)\right)\right)X,$$ (3)

where *σ* is the sigmoid activation function, *g* and *δ* are both 1D convolutional layers with kernel size 1, *W*_1_ and *W*_2_ are learnable weight matrices. *X* represents the input feature map with dimensions *C* × *H* × *W*, where *C* is the number of channels and *H*, *W* are the height and width, respectively.

### 2.4 Multiple coefficient class-specific residual attention block

**FIGURE 4:**
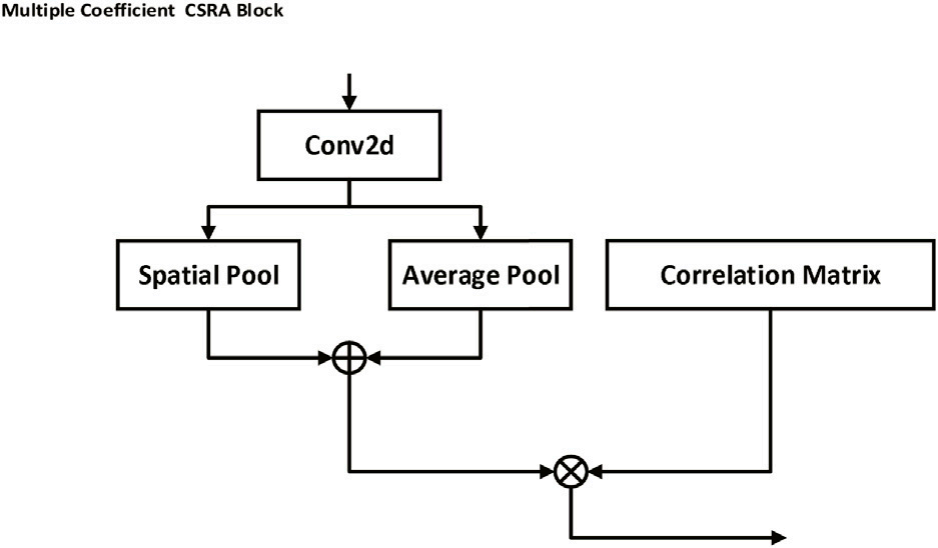
Multiple Coefficient CSRA block. This block uses trainable matrices as coefficients to capture the correlation between labels, thereby fostering a fusion of domain knowledge. By performing an average pooling operation on the input feature map and multiplying it by a weight matrix and a trainable matrix coefficient associated with a specific label, the block is able to generate prediction scores, facilitating label-specific attention and learning.

As shown in Figure 4, the Multiple Coefficient Class-specific Residual Attention (MC-CSRA) block is a variant of the CSRA block (Zhu and Wu, 2021) that incorporates trainable matrices as coefficients to learn the correlation between labels and achieve domain knowledge fusion, which can be expressed mathematically as:

$$\text{ Score }=AvgPool\left(x\right)\cdot W_{fc}\cdot {Coef}_{k},$$ (4)

where *x* represents the input feature map. *AvgPool* denotes the average pooling operation, which computes the global average feature representation of *x*. *W*_*fc*_ refers to the weight matrix of the fully-connected layer, which transforms the average feature representation to obtain the initial prediction scores. *Coef*_*k*_ represents the trainable matrix coefficient associated with the label, and *k* represents the index of the trainable matrix *Coef*_*k*_, which corresponds to a specific label.

## 3 Experiments and results

### 3.1 Dataset

**FIGURE 5:**
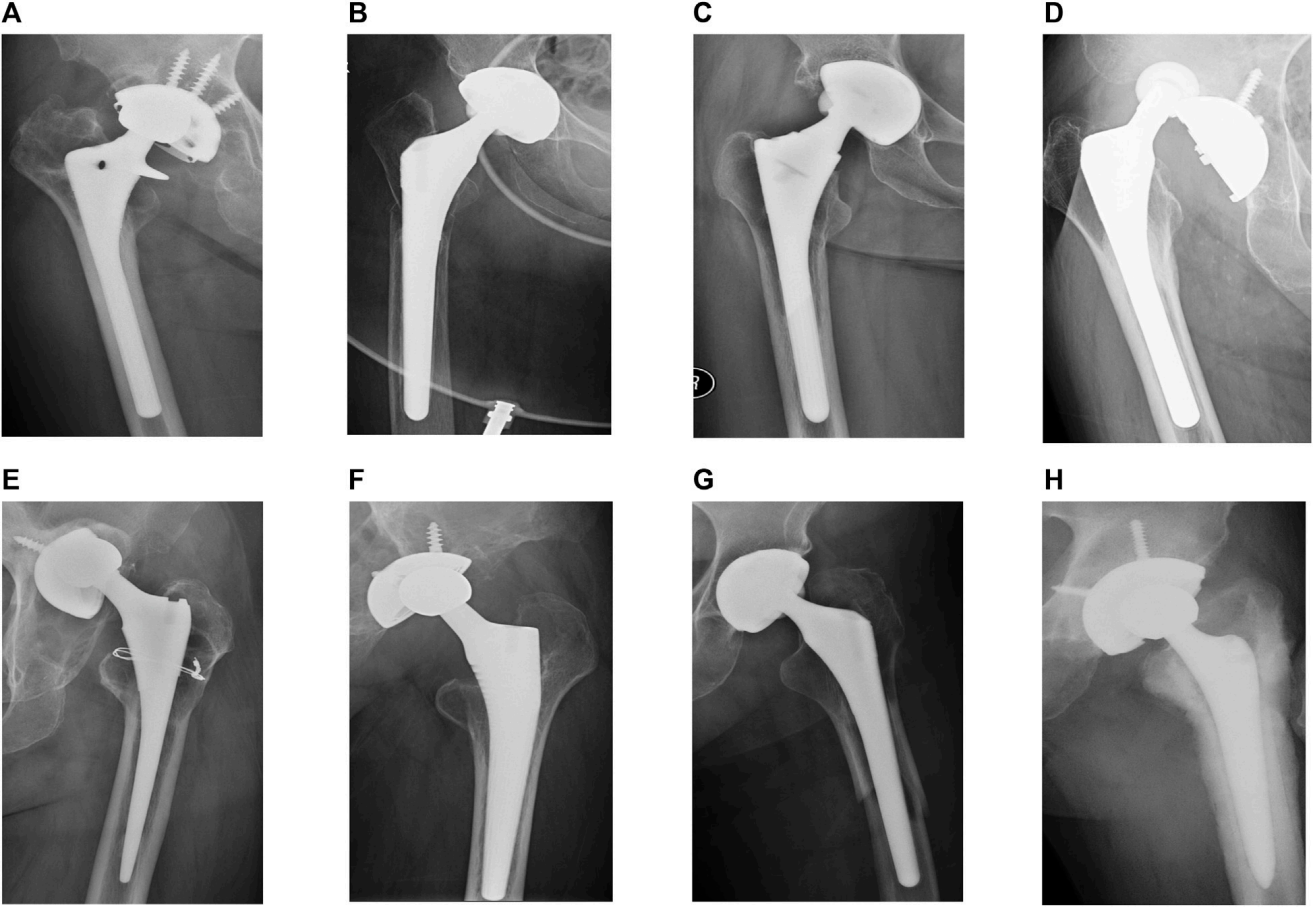
Examples of X-ray images of complications after total hip arthroplasty (THA) in our dataset cropped by our own trained YOLO-v5 model to preserve the periprosthetic images. Corresponding labels are labeled by an experienced orthopaedic surgeon as **(A)** loosening, periprosthetic osteolysis, and prosthetic wear; **(B)** periprosthetic fracture; **(C)** loosening; **(D)** dislocation; **(E)** loosening, periprosthetic osteolysis, and prosthetic wear; **(F)** periprosthetic osteolysis, and prosthetic wear; **(G)** loosening, periprosthetic fracture; and **(H)** loosening, periprosthetic infection.

Frontal and lateral X-ray images, MRI images, CT images, and clinical history data of patients that had reported complications after THR were collected by the author (GSJ) at Shanghai Ninth People’s Hospital. The dataset utilized in this work consisted of 443 preoperative X-ray images from patients scheduled to undergo revision after THR between 2014 and 2022. Data labeling was determined by an experienced orthopedic surgeon (ZJW) based on clinical history data and X-ray, CT, and MRI images. Each X-ray was meticulously annotated with seven 2-category indicators, corresponding to the presence or absence of aseptic loosening, periprosthetic osteolysis, periprosthetic fracture, dislocation, wear, infection, and other complications. During the data pre-processing phase, we initially trained an object recognition network based on YOLO-v5 (Jocher et al., 2022) and used it to crop the image surrounding the prosthesis. Subsequently, we normalized and converted the high-resolution X-ray images to JPEG format, which is required for inputting into the deep learning model. Figure 5 illustrates some typical X-ray images of postoperative complications after THA from our dataset.

In this study, we employed five-fold cross-validation to evaluate the performance of our proposed method. The dataset was randomly split into five equal folds. During each iteration of training and testing, four of these folds were used for training while the fifth was reserved for testing. This process was repeated five times, ensuring each fold served as the testing set once. The results across these five iterations were then averaged to produce a comprehensive model performance evaluation. The specific distribution of data in each fold is outlined in Table 1.

**TABLE 1:** Average number of images for each complication in training and testing sets across five-fold cross-validation.

Dataset	Number of X-ray images	Loosening	Osteolysis	Fracture	Dislocation	Wear	Infection	Other complications
Training Set	355	215	126	24	22	149	20	27
Testing Set	88	54	32	6	6	38	5	7
Total	443	269	158	30	28	187	25	34

### 3.2 Implementation details

The model developed in this study is based on the PyTorch framework. During the training phase, the model’s weights were updated using the Adam optimizer. The learning rate started at 0.0002 and was reduced by 10% after 5 epochs if the validation loss remained the same. The batch size of the model was set to 32 and the total number of epochs was 50. In the binary classification task, the dataset was divided into training, validation, and testing sets according to 6:2:2. To prevent overfitting, an early stopping strategy was used to terminates the training process before the model fully converged to prevent excessive memorization of the training data. We implement our method on Ubuntu with an NVIDIA GeForce RTX 3090 GPU.

### 3.3 Evaluation metrics

This study mainly used mAP (mean Average Precision) and F1 score as evaluation indicators to simultaneously identify multiple complications on a single x-ray image. mAP measures the average precision for each class and then takes the mean of these values to give an overall measure of performance. mAP is represented as

$$mAP=\frac{1}{C}\overset{C}{\underset{i=1}{\sum }}AP_{i},$$ (5)

where *C* is the number of classes and *AP*_*i*_ is the average precision for class *i*. The formula for average precision is:

$$AP_{i}=\frac{1}{n_{i}}\overset{n_{i}}{\underset{j=1}{\sum }}{\text{ Precision }}_{j}\cdot {\text{ rel }}_{j},$$ (6)

where *n*_*i*_ is the number of samples belonging to class *i*, *Precision*_*j*_ is the precision at the *j* ^*th*^ sample, and *rel*_*j*_ is an indicator variable that equals 1 if the *j* ^*th*^ sample belongs to class *i* and 0 otherwise.

The F1 score is expressed by Eq. 7, defined as the harmonic average of the precision and recall.

$$F1=\frac{2\cdot Precision\cdot \text{ Recall }}{\text{ Precision }+\text{ Recall }}$$ (7)

with the precision and recall calculated as

$$\text{ Precision }=\frac{TP}{TP+FP}$$ (8)

$$\text{ Recall }=\frac{TP}{TP+FN}$$ (9)

Given the challenges posed by class imbalance in our dataset—especially pertinent in the context of multi-classification problems—we adopted a weighted computational approach for more nuanced performance assessment. We introduce weighted variants of precision, recall, and the F1 score to better reflect the significance and distribution of each category within the dataset. This methodological refinement enhances the fairness and robustness of our performance evaluation, affording a more discerning analysis of the model’s capabilities.

The accuracy and F1 score were used to detect single complications on the X-ray images, which is a single-task binary classification problem. The accuracy measures the proportion of correctly classified samples out of the total number of samples, while the F1 score considers both the precision and recall, as previously discussed. The accuracy is be calculated by Eq. 10.

$$Accuracy=\frac{TP+TN}{TP+TN+FP+FN}$$ (10)

where *TP* is the true positives, *TN* is true negatives, *FP* is false positives, and *FN* is false negatives.

In addition to these performance metrics, we assessed the model’s complexity by considering both its spatial and temporal aspects. Such analysis allows the computational resources required by the model to be determined, which allows for comparison with other models and helps with selecting the most fitting model for specific tasks within different computational contexts. Spatial complexity was evaluated using the parameter count, as it is an apt representation of this aspect. A greater number of parameters denotes higher spatial complexity, a factor influenced by elements such as the network’s architecture, layer quantity, and each layer’s associated connection weights and biases. Temporal complexity, conversely, gauges the neural network model’s computational duration for either training or inference tasks. This is represented by the Floating-Point Operations Per Second (FLOPs) metric, which indicates the quantity of addition and multiplication operations conducted by the model. A higher FLOP value signifies greater temporal complexity. Elements such as the network’s architecture, the number of layers, and the size of the inputs all contribute to the computation of FLOPs. To provide a more tangible understanding of our model’s temporal complexity, we also determined its actual inference time.

### 3.4 Benchmark model comparison

Our study assessed the efficacy of various benchmark models at image classification tasks using our dataset. Table 2 illustrates the comparative average performance of these benchmark models under five-fold cross-validation. ResNet18 conspicuously stood out with an mAP of 31.0% and an F1 score of 35.7%, both of which were the highest among the evaluated models. This indicates ResNet18’s superior performance in achieving a well-rounded classification on our dataset. While DenseNet121 marginally surpassed ResNet18 in precision with a score of 50.1%, it did not fare as well in the other metrics. On the other hand, MobileNet_v3 exhibited the highest recall at 36.8%. However, a high recall without balanced precision can lead to an increased number of false positives, which may not be ideal for clinical settings.

**TABLE 2:** Performance comparison of benchmark methods using five-fold cross-validation: average results.

Method	mAP (%)	Precision (%)	Recall (%)	F1 score (%)	Params (×10^6^)	FLOPs (×10^6^)
ResNet18 He et al. (2016)	31.0	48.3	34.0	35.7	11.18	1.82
ResNet50	29.3	30.6	20.8	23.0	23.52	4.13
ResNet101	29.2	25.3	24.7	25.0	42.52	7.86
DenseNet121 Huang et al. (2017)	30.7	50.1	27.6	35.3	6.96	2.90
DenseNet161	29.9	39.0	21.3	26.4	26.49	7.84
GoogLeNet Szegedy et al. (2015)	30.2	37.9	30.6	32.9	5.61	1.51
MobileNet v3 Howard et al. (2019)	27.3	22.0	36.8	27.4	1.53	61.46
EfficientNet Tan and Le, (2019)	29.3	41.4	30.0	32.1	4.02	413.87

The ResNet18 model also demonstrated a clear balance between computational efficiency and performance. The model’s parameter count, indicative of its spatial complexity, is notably lower than that of ResNet50 and ResNet101, while being comparable to models such as DenseNet121 and GoogLeNet. This low spatial complexity corresponds to low memory usage during both training and inference stages, positioning ResNet18 as a cost-effective choice, especially within resource-limited settings. Furthermore, the lower FLOPs of ResNet18, indicative of its temporal complexity, are considerably lower than most of the alternative models. This suggests ResNet18 requires fewer computational resources for either an inference or a training task, promoting faster training and inference times, which are essential characteristics for applications requiring a quick response.

In conclusion, although ResNet18 was not the top performer in all metrics, the model was capable of a robust classification while maintaining lower spatial and temporal complexity compared to the other evaluated models. This balance between performance and computational efficiency influenced our selection of ResNet18 as the backbone for our methodology and served as a benchmark for assessing the effectiveness of the proposed classification methods. This enabled us to build on a strong model foundation while optimizing the use of computational resources.

### 3.5 Results of ablation study

The comprehensive ablation study under five-fold cross-validation clearly demonstrated the effectiveness of our proposed model (ResNet18 + GFS + CFS + MC-CSRA). As detailed in Table 3, the model performed exceptionally well across all classification metrics tested, achieving an mAP of 34.6% and an F1 score of 42.9%. Notably, each individual component–the Global feature stream (GFS), Channel feature stream (CFS), and the Multiple Coefficient CSRA block (MC-CSRA) – made a significant contribution to this augmented performance.

**TABLE 3:** Ablation study results using five-fold cross-validation: average results.

Method	mAP (%)	Precision (%)	Recall (%)	F1 score (%)	Params (×10^6^)	FLOPs (×10^6^)	Inference (*s*)
ResNet18 (Baseline)	31.0	48.3	34.0	39.9	11.18	1.82	0.0019
RestNet18 + GFS	33.3	55.1	43.7	48.7	20.69	3.47	0.0025
RestNet18 + GFS + MC-CSRA	33.5	56.7	42.5	40.3	20.69	3.47	0.0025
RestNet18 + CFS	31.8	53.8	37.3	44.0	11.25	2.06	0.0019
RestNet18 + CFS + MC-CSRA	33.5	57.8	38.7	46.4	11.25	2.06	0.0019
RestNet18 + GFS + CFS	32.1	55.1	40.7	46.8	20.72	3.59	0.0029
RestNet18 + GFS + CFS + MC-CSRA (Proposed)	34.6	57.3	34.3	42.9	20.72	3.59	0.0029

GFS, Global feature stream; CFS, Channel feature stream; MC-CSRA, Multiple Coefficient CSRA block.

Incorporating GFS and CFS into the baseline ResNet18 model validated the importance of these features in enhancing model performance. When the GFS component was integrated, the mAP increased to 33.3% and the F1 score rose to 48.7%, which indicates that GFS is pivotal in capturing broader, global features. In parallel, the introduction of a CFS component boosted the mAP to 31.8% and the F1 score to 44.0%, underscoring CFS’s capacity in extracting local, nuanced features, subsequently enhancing classification efficacy. The addition of an MC-CSRA block further amplified the model’s performance. For the ResNet18 + GFS model, the mAP slightly rose to 33.5%, however, the F1 score saw a decrease to 40.3%. The ResNet18 + CFS model, on the other hand, experienced improvements with the mAP advancing to 33.5% and the F1 score surging to 46.4%. Such advancements emphasize the critical role of the MC-CSRA block in elevating the model’s discriminative prowess.

Further ablation studies were performed to validate the robustness of the proposed modules against different backbone models. These studies used ResNet50, DenseNet121, and Densenet161 as the backbones, as detailed in the Supplementary Tables S1–S3. The enhanced performance of the ResNet50-based model is illustrated in Supplementary Table S1. As was observed with the ResNet18 model, the inclusion of the Global Feature Stream (GFS) and the Channel Feature Stream (CFS) bolstered the ResNet50 model’s performance. The final addition of our proposed Multiple Coefficient CSRA block (MC-CSRA) further augmented the mean average precision (mAP) and F1 score, reaching 31.5% and 33.7%, respectively, thus demonstrating the strong contribution of each component to the model’s overall performance. DenseNet121-based and Densenet161-based models were similarly evaluated, with results outlined in Supplementary Tables S2, S3. The performance of these models also improved after the inclusion of the GFS, CFS, and MC-CSRA block, reaching a final mAP of 32.5% and 33.2% and an F1 score of 34.6% and 37.7%, respectively.

While improving performance, our proposed model (ResNet18 + GFS + CFS + MC-CSRA) also maintained a reasonable model complexity. The number of parameters slightly increased to approximately 20.72 million, while the FLOPs rose to around 3.59 million, thus achieving a well-balanced trade-off between performance and computational efficiency.

In addition, we have also conducted an analysis of the prediction performance of different models for specific labels. Table 4 showcases the accuracy and F1 scores of these models in predicting respective complications. The ResNet18+GFS+CFS configuration outperformed in “Osteolysis” with an accuracy of 68.2%. The “Loosening” label saw the ResNet18+CFS model excel, posting an accuracy of 61.6%. While accuracy for “Fracture” remained consistent across models, the ResNet18+GFS+CFS+MC-CSRA edged out in F1 at 90.5%. For “Dislocation” and “Wear” complications, the ResNet18+GFS and ResNet18+GFS+MC-CSRA models respectively delivered peak performances. In the “Infection” category, ResNet18+GFS+CFS+MC-CSRA achieved an unmatched accuracy of 94.6% and F1 score of 92.4%. Under the “Others” label, the same model continued to lead, registering an accuracy of 93.0%.

**TABLE 4:** Comparative performance of various models in identifying each post-surgical complication using five-fold cross-validation: average results (%).

Model	Osteolysis		Loosening		Fracture		Dislocation		Wear		Infection		Others	
	Acc	F1	Acc	F1	Acc	F1	Acc	F1	Acc	F1	Acc	F1	Acc	F1
ResNet18	62.7	53.2	58.0	55.9	93.3	90.0	93.7	90.6	60.0	57.4	94.4	91.6	92.3	88.6
ResNet18 + GFS	62.5	58.8	59.8	59.1	93.3	90.0	94.1	91.6	62.3	61.1	94.4	91.6	92.8	89.6
ResNet18 + GFS + MC-CSRA	63.2	60.5	56.7	55.6	93.3	90.0	94.1	91.6	63.0	60.2	94.4	91.6	92.8	89.6
ResNet18 + CFS	66.1	58.5	61.6	61.2	93.3	90.0	93.7	90.6	62.5	59.8	94.4	91.6	92.3	88.6
ResNet18 + CFS + MC-CSRA	64.5	58.7	60.6	59.1	92.8	90.5	93.7	91.0	59.3	56.9	94.4	91.6	92.8	89.6
ResNet18 + GFS + CFS	68.2	62.5	60.7	60.4	93.3	90.0	93.7	90.6	61.8	59.9	94.4	91.6	92.3	88.6
ResNet18 + GFS + CFS + MC-CSRA	65.7	57.9	54.0	52.7	93.5	90.5	94.1	91.6	58.2	53.9	94.6	92.4	93.0	90.3

GFS, Global feature stream; CFS, Channel feature stream; MC-CSRA, Multiple Coefficient CSRA block; Acc, Accuracy; F1, F1-score.

In conclusion, our model leveraged the strengths of GFS, CFS, and the MC-CSRA block to achieve robust performance. The notable improvements in mAP, F1 scores, and specific complication accuracies underline its effectiveness. Importantly, these results were achieved while striking a balance between performance and computational efficiency.

### 3.6 Results of binary classification tasks and external datasets

Our proposed multi-branch neural network model (ResNet18 + GFS + CFS) exhibited superior performance over the baseline model (ResNet18) at binary classification tasks, which demonstrated the effectiveness of the additional modules. This performance was assessed on two datasets: a labeled loosening subset of our multi-labeled dataset and an external loosening dataset (Rahman et al., 2022).

Table 5 details the binary classification performance on our internal dataset. The proposed model significantly outperformed the baseline ResNet18, achieving an accuracy of 88.1% and an F1 score of 89.7%. In contrast, the baseline model yielded an accuracy of 71.4% and an F1 score of 84.0%, highlighting the substantial improvement attained by incorporating the Global feature streams (GFS) and Channel feature streams (CFS) into the baseline model.

**TABLE 5:** Comparison of the binary classification performance of different methods on loosening subset of our dataset (%).

Method	Accuracy	Precision	Recall	F1-score
ResNet18	71.4	84.0	72.4	77.8
ResNet18 + GFS + CFS (Proposed)	88.1	92.9	89.7	91.2
Loppini et al. (2022)	78.6	91.7	75.9	83.0
Lau et al. (2022)	73.8	85.7	72.4	79.2

GFS, Global feature stream; CFS, Channel feature stream.

Comparative experiments against methods proposed in (Lau et al., 2022; Loppini et al., 2022) further highlighted the robustness of our model, as seen in Table 6. Our approach exhibited noteworthy gains, surpassing the performance of these cited methods on the loosening subset of our dataset, both in terms of accuracy and F1-score. The results on the external loosening dataset, as shown in Table 6, reveal a more nuanced picture. While our model outperformed both the method by Loppini et al. and Lau et al. with accuracy scores of 54.5% and 58.0%, respectively, it fell slightly short of the HipXNet (Rahman et al., 2022) performance. The HipXNet model achieved an accuracy of 96.1%, with our model trailing at 92.9%. Given that HipXNet employs a more complex, stacked CNN model, we perceive this narrow performance differential as acceptable.

**TABLE 6:** Comparison of the binary classification performance of different methods on external loosening dataset (%).

Method	Accuracy	Precision	Recall	F1-score
ResNet18	56.8	67.3	62.5	64.8
ResNet18 + GFS + CFS (Proposed)	92.9	96.4	93.1	94.7
Loppini et al. (2022)	54.5	73.7	44.6	55.6
Lau et al. (2022)	58.0	73.2	53.6	61.9
HipXNet Rahman et al. (2022)	96.1	96.4	96.4	96.7

GFS, Global feature stream; CFS, Channel feature stream.

In conclusion, the binary classification tests performed on both the internal and external datasets corroborate the effectiveness of our proposed multi-branch neural network model. The substantial improvements over the baseline model on both datasets underscore the contribution of the Global and Channel feature streams (GFS and CFS) towards enhancing the model’s performance. The model’s robust performance, even in comparison to specialized methods such as HipXNet on external datasets demonstrates its potential for use in diverse and complex classification tasks.

## 4 Discussion

Diagnosing complications after THA can be challenging in clinical practice due to the complexity and variability of X-ray manifestations, as well as the potential overlap between symptoms and other diseases. However, using an artificial intelligence system to assess the radiographs can potentially improve the speed and accuracy of the diagnosis. This study presents a multi-branch network that was capable of accurately detecting complications following THA. By utilizing multiscale and multilevel network models, different image features can be effectively captured, yielding a better performance than conventional methods. The assessments in this study were carried out using a comprehensive multi-label dataset of complications following THA, composed of high-quality X-ray images of hip prosthesis failures. Furthermore, this study demonstrated the effectiveness of domain prior fusion, showing that combining domain-specific information can drastically improve model performance.

The ablation studies presented in this report, performed across both multi-label and binary classification tasks, clearly show that the proposed ResNet18+GFS+CFS model outperforms the baseline ResNet18. The superior performance enhancement is primarily due to the seamless integration of GFS and CFS, allowing the module to capture both global and local features. This, in turn, produces a more comprehensive feature map of THR complications. These findings echo previous research (Xie et al., 2021), substantiating the claim that a judicious combination of global and local features substantially boosts image classification performance. In particular, this highlights the importance of both fine-grained local features and global context for discerning complex patterns, especially in the realm of medical imaging. This study also serves as a proof of concept that existing models like ResNet18 can be refined by integrating task-specific components, which can potentially be generalized to various image-based medical diagnostic tasks.

From clinical practice experience, the authors observed that there may be certain underlying correlations between the occurrence of postoperative complications in the same patient during the same period. For example, post-THA infections typically occur independently from other complications, whereas periprosthetic osteolysis can lead to aseptic loosening. Therefore, we hypothesized a correlation between the labels. To exploit the potential relationships between these label classes, we devised an MC-CSRA (multi-label classification with class-specific regional attention) block that enabled the model to learn correlations between different labels, thereby improving the prediction accuracy. Ablation experiments also demonstrated a considerable improvement in overall performance after integration of the MC-CSRA module. These findings are consistent with previous research (Muralidhar et al., 2018; Xie et al., 2021), which suggests that integrating domain knowledge can improve the efficiency and accuracy when data is scarce or noisy. In contrast, rather than introducing domain knowledge directly, we designed a module that allows the model to independently acquire or focus on relevant domain knowledge, even if it appears to be easily understandable to humans, which can greatly improve the performance of the model.

Building upon this, our findings from the five-fold cross-validated ablation experiments offer valuable insights. The incorporation of the MC-CSRA block unmistakably enhanced the model’s overall mAP. It also heightened the predictive accuracy for complication classes with fewer images, albeit with a mild reduction in the predictive acumen for more image-abundant complication classes. This trend can be traced back to the MC-CSRA mechanism’s design, which accentuates inter-class dynamics. By earnestly seeking out correlations amongst diverse labels and amalgamating domain-specific knowledge, the mechanism might amplify the focus on infrequent complications. This shift can occasionally temper the performance for more dominant complications, exemplified by ‘loosening’. Given today’s medical imaging milieu marked by pronounced data imbalances, the MC-CSRA’s approach provides a meaningful way to balance performance across varied categories. While the MC-CSRA block has shown potential in enhancing model performance for classes with fewer images, it is essential to note the observed decrement in performance for classes with abundant data. This deviation is not negligible, especially when considering the clinical relevance of categories like ‘loosening’ that are often key indicators for postoperative revision surgery. This effect is likely attributable to the attention shift induced by the MC-CSRA mechanism, causing the model to dilute its focus on dominant yet clinically significant classes. Therefore, the adoption of the MC-CSRA block comes with an implicit trade-off that practitioners should consider carefully based on the clinical objectives. Future work should explore mechanisms for tuning the MC-CSRA block to mitigate the observed performance decrements in dominant classes, potentially through weighting schemes or hybrid attention models. In a clinical setting where both prevalent and infrequent complications are of significant concern, the choice to incorporate MC-CSRA must be clinically justified, and the limitations carefully weighed against its advantages.

Our study, to the best of our knowledge, is the first to use deep learning for the automatic detection of multiple complications in X-ray images. Prior research in the field, such as the works by Shah et al. (2020), Rahman et al. (2022), Loppini et al. (2022), and Lau et al. (2022) , showed promising results but often focused on identifying specific types of complications like loosening or dislocation. Our approach broadens the horizon by being able to detect a wider spectrum of complications. Specifically, our model is not only capable of identifying different types of complications but can also automatically flag multiple complications. Unlike previous approaches, it does not depend on historical or demographic data from patients or specific information about the diagnosed complications. The proposed model can pinpoint complications on X-ray images, whether they are in the anterior or lateral view.

Our research is a significant step forward from previous attempts at applying multi-label classification to X-ray imaging (Xu et al., 2020; Wang et al., 2021). The approach drew from similar concepts using multi-label classification but innovatively applied it to THA complications. Moreover, our method uniquely allows for the interrelationships between different complications to be understood, whereby the MC-CSRA block is intentionally designed to learn the correlations between distinct labels. This enhances not only the prediction accuracy but also offers insightful observations into the interconnected nature of postoperative complications. Our model operates independently, identifying complications solely from X-ray images regardless of their orientation, which underscores the robustness of our approach. We believe these advancements clearly demonstrate the novelty of our work, extending the boundaries of deep learning applications in medical imaging and setting a new standard for the automatic detection of multiple THA complications.

Although our study yielded positive results, it also has some limitations. Firstly, the number of images of some complications in our dataset, such as infections and fractures, is limited, and even with model improvements, the accuracy may need further validation for clinical use. Secondly, our imaging data originated from preoperative images of patients scheduled for THA revision surgery. Therefore, the algorithm has not been assessed in patients that have not been diagnosed with a complication or if symptoms are not severe enough to warrant surgery. The accuracy, sensitivity, and specificity values for the proposed model may not be applicable to this population. Lastly, further studies are required to authenticate the generalisability of our proposed multi-branch network using larger datasets across multiple institutions.

Future research in this area should aim to broaden the scope of the dataset and incorporate a more comprehensive range of complications for more precise results that are applicable to a wider population. It is pertinent to accurately identify complications on X-ray images for effective preoperative planning of THA revision surgery as this helps delineate appropriate surgical access, implant selection and surgical techniques. Therefore, an encouraging strategy would be to integrate deep learning methods for identifying complications during the preoperative planning of THA revision surgery.

In conclusion, this study presents a pioneering approach to using a multi-branch network based on X-ray images to identify complications following THA. The findings of this study highlight the efficacy of using deep learning techniques for detecting complications as well as the benefits of leveraging domain-specific prior knowledge to enhance the model’s performance. The findings of this study serve as a foundation for further research in diagnosing complications after THA. Future research could focus on constructing larger datasets with different complications to improve the accuracy and robustness of the model.

## Data Availability

The datasets presented in this article are not readily available because a portion of the dataset will be made publicly available in the future, while access to the full dataset will only be available after contacting the corresponding author and submitting the appropriate request to be approved by the author’s institution. Requests to access the datasets should be directed to JZ, zjw_ys@163.com.
