# Supplementary material for: A multi-branch network to detect post-operative complications following hip arthroplasty on X-ray images
Source: Front Bioeng Biotechnol. 2023 Sep 28;11:1239637. doi: 10.3389/fbioe.2023.1239637 (PMC10569301; doi:10.3389/fbioe.2023.1239637)
Supplement: Supplementary file 1 [file Table1.pdf]

Supplementary Material

1 SUPPLEMENTARY TABLES AND FIGURES

Table S1. Results of ablation experiments when using ResNet50 as a backbone for improvement(%).

Method	mAP	Precision	Recall	F1-score
ResNet50	29.3	30.6	20.8	23.0
ResNet50 + GFS	30.04	36.1	22.5	26.0
ResNet50 + GFS + MC-CSRA	32.0	49.5	30.8	36.3
ResNet50 + CFS	30.3	35.9	26.0	28.9
ResNet50 + CFS + MC-CSRA	31.3	47.6	32.2	40.0
ResNet50 + GFS + CFS	30.4	41.1	26.3	30.3
ResNet50 + GFS + CFS + MC-CSRA	31.5	50.1	29.1	33.7

GFS: Global feature stream. CFS: Channel feature stream. MC-CSRA: Multiple Coefficient CSRA block.

Table S2. Results of ablation experiments when using DenseNet121 as a backbone for improvement(%).

Method	mAP	Precision	Recall	F1-score
DenseNet121	30.7	50.1	27.6	33.0
DenseNet121 + GFS	32.3	56.4	42.4	44.5
DenseNet121 + GFS + MC-CSRA	30.7	41.0	28.8	31.6
DenseNet121 + CFS	32.3	53.0	46.3	47.6
DenseNet121 + CFS + MC-CSRA	33.0	57.4	42.7	41.0
DenseNet121 + GFS + CFS	32.3	52.7	34.6	43.0
DenseNet121 + GFS + CFS + MC-CSRA	32.5	50.2	32.0	34.6

GFS: Global feature stream. CFS: Channel feature stream. MC-CSRA: Multiple Coefficient CSRA block.

Table S3. Results of ablation experiments when using DenseNet161 as a backbone for improvement(%).

Method	mAP	Precision	Recall	F1-score
Densenet161	29.9	39.0	21.3	26.4
DenseNet121 + GFS	31.6	48.9	42.4	43.2
Densenet161 + GFS + MC-CSRA	32.3	42.5	29.5	33.0
Densenet161 + CFS	32.5	53.8	42.0	44.8
Densenet161 + CFS + MC-CSRA	32.5	54.0	32.5	40.4
Densenet161 + GFS + CFS	32.4	54.1	45.3	45.1
Densenet161 + GFS + CFS + MC-CSRA	33.2	46.3	37.4	37.7

GFS: Global feature stream. CFS: Channel feature stream. MC-CSRA: Multiple Coefficient CSRA block.
